# Delirium and other neuropsychiatric manifestations of COVID-19 infection in people with preexisting psychiatric disorders: a systematic review

**DOI:** 10.1186/s13256-021-03140-6

**Published:** 2021-12-13

**Authors:** Emma A. van Reekum, Tea Rosic, Anjali Sergeant, Nitika Sanger, Myanca Rodrigues, Reid Rebinsky, Balpreet Panesar, Eve Deck, Nayeon Kim, Julia Woo, Alessia D’Elia, Alannah Hillmer, Alexander Dufort, Stephanie Sanger, Lehana Thabane, Lawrence Mbuagbaw, Zainab Samaan

**Affiliations:** 1 Department of Psychiatry and Behavioural Neurosciences, Clinician Investigator Program, McMaster University, Hamilton, Canada; 2 Department of Psychiatry and Behavioural Neurosciences, McMaster University, Hamilton, Canada; 3 Michael G. DeGroote School of Medicine, McMaster University, Hamilton, Canada; 4 Medical Science Graduate Program, McMaster University, Hamilton, Canada; 5 Health Research Methodology Graduate Program, McMaster University, Hamilton, Canada; 6 Neuroscience Graduate Program, McMaster University, Hamilton, Canada; 7 Health Sciences Library, McMaster University, Hamilton, Canada; 8 Department of Health Research, Evidence and Impact, McMaster University, Hamilton, Canada; 9 St. Joseph’s Healthcare Hamilton, 100 West 5th Street, Hamilton, ON L8N 3K7 Canada

**Keywords:** Delirium, Mental disorders, COVID-19, Internal medicine, Pandemic, Psychiatric disorder, Systematic review, Case reports

## Abstract

**Background:**

Psychiatric disorders increase risk of neuropsychiatric disease and poor outcomes, yet little is known about the neuropsychiatric manifestations of COVID-19 in the psychiatric population. The primary objective is to synthesize neuropsychiatric outcomes of COVID-19 in people with preexisting psychiatric disorders.

**Methods:**

Data were collected during an ongoing review of the impact of pandemics on people with existing psychiatric disorders. All study designs and gray literature were included. Medline, PsychInfo, CINAHL, EMBASE, and MedRx were searched from inception to September 1 2020. Risk of bias was assessed using a published tool that can accommodate all study types. Two independent authors screened the studies and extracted data. Data were narratively synthesized, as there were insufficient data to meta-analyze. Evidence was appraised according to GRADE.

**Results:**

Four case reports were included, comprising 13 participants from three countries. Many large-sample, relevant papers were omitted for not reporting psychiatric history, despite reporting other comorbidities. Included participants (*n* = 13) were hospitalized with COVID-19 and appeared to meet criteria for delirium. Myoclonus, rigidity, and alogia were also reported. The most commonly reported preexisting psychiatric diagnoses were mood disorders, schizophrenia, and alcohol use disorder.

**Conclusions:**

People with preexisting psychiatric disorders may experience delirium, rigidity, myoclonus, and alogia during COVID-19 infection; although higher quality and longitudinal data are needed to better understand these phenomena. Relevant COVID-19 literature does not always report psychiatric history, despite heightened neuropsychiatric vulnerability within this population.

*Trial Registration:* PROSPERO (CRD42020179611).

**Supplementary Information:**

The online version contains supplementary material available at 10.1186/s13256-021-03140-6.

## Background

### Rationale

Respiratory viruses can also infect the central nervous system (CNS), and coronaviruses are among those shown to have neuro-invasive properties [1]. Human studies during prior coronavirus outbreaks found an array of neuropsychiatric sequelae associated with infection. For instance, autopsy studies revealed associations between SARS-CoV infection, cerebral edema, and meningeal vasodilation [2]. Clinically, neuropsychiatric symptoms were reported in up to 20% of critical care patients with MERS-CoV, of which confusion was most common [3]. A systematic review of coronaviruses found delirium to be the most prevalent neuropsychiatric sequelae in both MERS-CoV and SARS-CoV, occurring in almost 30% of infected patients [4].

Emerging data suggest the novel coronavirus, SARS-CoV-2, is also neurotropic [5]. Various mechanisms for neuropathogenesis have been proposed including blood–brain barrier disruption [6], neuro-infiltration via immune cells [5] or vascular endothelium [7], as well as entry via the olfactory nerve [5, 7]. The CNS could also be vulnerable through indirect mechanisms including cerebral hypoxia from lung damage, metabolic derangements from renal injury, hypercoagulability precipitating thromboembolic events [5], and adverse drug events in COVID-19 management [7].

As COVID-19 cases continue to rise across the globe, associated CNS manifestations are increasingly recognized in the literature. Initial signals of CNS involvement in SARS-CoV-2 emerged from reports of encephalitis, ageusia, and anosmia during acute infection [8]. An early systematic review of 409 patients with CNS complications of COVID-19 found the most common acute neurological and neuropsychiatric symptoms to be headache (16.8%), dizziness (13.9%), and delirium (11.2%) [9]. In keeping with other coronaviruses, delirium may be the most frequent neuropsychiatric manifestation of COVID-19 [10, 11], occurring in 37–42% of hospitalized patients [12, 13] and 3% of total cases [14]. Emerging evidence suggests delirium may also be an important prognostic factor, predicting poorer outcomes from COVID-19 [15].

Neuropsychiatric syndromes are frequently ill-defined and with considerable inconsistencies in the terminology reported in the literature. Several terms are employed to report the same phenomenon. For instance, according to well-utilized diagnostic criteria [16], delirium is an acute syndrome of inattention and impaired awareness accompanied by a change in cognitive function; however, this disorder is also described as “CNS dysfunction,” “impaired awareness,” “attentional difficulties,” “lethargy,” “confusion,” and “encephalopathy” in the literature [4,^ 7^,^ 10^, 17]. To prioritize consistency in reporting, we aligned our definition of neuropsychiatric outcomes with the earliest review [4] of COVID-19 neuropsychiatric sequelae and, similarly, chose not to focus on primarily neurological conditions such as encephalitis and stroke.

The pathophysiology for neuropsychiatric syndromes is not well understood. However, people with psychiatric disorders appear to have an elevated risk for many reasons, such as underlying pathophysiological differences, as well as risks due to psychotropic medications and polypharmacy [18–22]. Given the heightened vulnerability of people with psychiatric disorders to neuropsychiatric syndromes, it is important to study these phenomena in this population separately. To our knowledge, there is no synthesis on the neuropsychiatric manifestations of COVID-19 infection in people with psychiatric disorders.

### Objectives

The primary aim of this review is to provide a synthesis of the neuropsychiatric sequelae of COVID-19 in patients with psychiatric disorders. Secondly, we will examine vulnerability for neuropsychiatric manifestations of COVID-19 by type of psychiatric disorder. As an exploratory aim, we will synthesize the management approaches used to treat neuropsychiatric disease in our population of interest, due to the variety of behavioral and pharmacotherapies that can employed.

## Methods

### Eligibility criteria

This review was written in accordance with the PRISMA reporting guidelines [23] (see Additional file 1: Table S1. for research checklist). The protocol for our systematic review has been published [24]. This systematic review included multiple aims that has yielded multiple papers. The present paper presents data from just one aim, which included studies that met the following criteria: (1) included participants with a psychiatric disorder that predated the COVID-19 pandemic, (2) reported neuropsychiatric manifestations of COVID-19 infection, and (3) had a COVID-19 infection as determined by either polymerase chain reaction (PCR), antibody test, or strong clinical suspicion. In keeping with Rogers *et al*. [4], we formulated our definition of “neuropsychiatric” to include conditions that can impact mental status and psychiatric disorder manifestation, such as: delirium, sleep difficulties, apathy, catatonia, and amnesia. We included studies that reported on a population with at least one of the following preexisting diagnoses according to the Diagnostic and Statistical Manual of Mental Disorders (DSM-5) [16]: attention deficit hyperactivity disorder, autism spectrum disorder, schizophrenia and other psychotic disorders, bipolar and depressive disorders, anxiety and obsessive–compulsive and related disorders, trauma and stressor-related disorders, somatic symptom and related disorders, feeding and eating disorders, substance use disorders, and personality disorders. We did not limit our search strategy to specific diagnostic criteria for psychiatric disorders and accepted a range of diagnostic strategies including clinician diagnosed, self-reported history, medical charts, or symptom scales. We chose not to include neurocognitive disorders because of the challenges of distinguishing COVID-19 neuropsychiatric effects from underlying cognitive deficits. We included all study designs, including gray literature, to provide as comprehensive a review as possible. Papers were ineligible if they did not present data for our population of interest (people with preexisting psychiatric disorders who had COVID-19 infection) or if an outcome of interest (neuropsychiatric sequelae) was not reported.

### Information sources

The search period covered the years from the databases’ inception to September 1 2020. The databases Medline, EMBASE, PsychInfo, and CINAHL were searched on the platforms Ovid and EBSCOhost. Because of the ongoing nature of the COVID-19 pandemic and emphasis on rapid knowledge dissemination, gray literature was also searched manually on MedRx.

### Search strategy

Our search strategy was designed in consultation with a health sciences librarian (SS) and with co-authors who have expertise in systematic reviews, knowledge synthesis, and psychiatric disorders. The search strategy assesses the impact of all pandemics and epidemics (including COVID-19) throughout history on people with psychiatric disorders. Our search strategy can be found in Additional file 2: Table S2 of the supplementary material. The only limits applied to our search strategy were language (English only) and date (on or before September 1 2020). We conducted the search manually; no artificial intelligence or natural language processing tools were used. Preferred reporting items for systematic reviews and meta-analyses (PRISMA) 2020 guidelines were followed and fulfilled [25].

### Selection process

All titles, abstracts, and full texts were screened in duplicate. The two reviewers consulted a senior author to resolve any conflicts that arose. The Covidence platform [26] for systematic review management was used for the screening and extraction processes, however, no machine learning tools were utilized.

### Data collection process

Data were extracted independently and in duplicate from January to March 2021 by two authors (EvR, TR). Differences were resolved by consensus between the two authors. Study investigators were not contacted, and no automation, extraction, or translation tools were utilized.

### Data items (outcomes)

Our outcomes were neuropsychiatric manifestations of COVID-19 infection, which in concordance with our search strategy included: delirium (including terms such as confusion, amnesia, dementia), sleep-related outcomes (including fatigue, tiredness, insomnia, somnolence, hypersomnolence, parasomnia), movement disorders, irritability (including agitation, aggression), apathy (including indifference), disinhibition, catatonia, hallucinations, and delusions. Given data on the prognostic importance of delirium [27] and its prevalence in COVID-19 infection [11], delirium was considered the most important neuropsychiatric outcome. Given the lack of literature on this topic, we did not specify a timeframe for these outcomes, and deemed studies to be eligible as long as the outcomes occurred concurrently with a confirmed or suspected COVID-19 infection. When papers described features consistent with our definition of delirium as above (e.g., confusion, encephalopathy), we made the assumption that the patient had delirium.

### Data items (other variables)

Other data were extracted, including sample size, preexisting psychiatric disorder type(s), country of study, management (behavioral and pharmacotherapy), and eventual patient disposition (improved, recovered, palliative, deceased). No assumptions were made about unclear variables from the studies and no tools to inform data collection were used. When papers reported data for patients without a psychiatric history, data were only extracted for the psychiatric population, specifically.

### Study risk of bias assessment

To our knowledge there is no universally accepted quality tool for case series and reports. As such, we selected a proposed framework for assessing the risk of bias in case reports that aligns with previously validated tools [28]. This tool consists of eight dichotomous questions that are grouped into four domains including: selection, ascertainment, causality, and reporting. Scoring can provide an aggregate of a maximum of eight points, however, the superior approach is to provide an overall judgment based on the specific values deemed most relevant to the review. Authors EvR and TR determined that “ascertainment of exposure” and whether “alternative causes that may explain the observation were ruled out” were the two most important characteristics to evaluate quality. The authors then independently assessed risk of bias and discussed disagreements to reach consensus.

### Effect measures

We considered the presence or absence of neuropsychiatric manifestation in the included studies as a binary outcome.

### Synthesis methods (eligibility for synthesis)

A meta-analysis was not possible according to our protocol [24] given the heterogeneous literature and the limited number of studies that met the inclusion criteria.

### Synthesis methods (preparing for synthesis)

We did not prepare the data for summary statistics as the included studies were not feasible for meta-analysis.

### Synthesis methods (statistical synthesis methods)

As a meta-analysis was not possible, a narrative synthesis was chosen as the ideal synthesis approach for our study findings. Our narrative synthesis was guided by prior recommendations [28, 29], and involved organizing the data in tabular form for a preliminary synthesis. Second, we grouped studies by the following themes: (1) the type of neuropsychiatric manifestation, (2) the preexisting psychiatric disorder; (3) the participants’ age; and (4) the reported management approaches and challenges that are specific to the psychiatric population.

### Reporting bias assessment

No specific tools were utilized to assess risk of bias because of missing results. Study authors were not contacted to confirm relevant information.

### Certainty assessment

The GRADE framework was used to assess confidence in the body of evidence presented in this review. Two authors independently assessed certainty and resolved disagreements by discussion. The results from the certainty assessments are reported in the Summary of Findings table (Table 1.).

**Table 1:** Summary of Study Findings

ID Country Sample size (*n*)	Case# Diagnosis Age Sex	Description of neuropsychiatric outcomes	Disposition	GRADE
Beach 2020 United States *n* = 4	(1) AUD—remitted, NCD 76 years Female	Admitted with aggression, paranoia, alogia, and abulia. On examination had myoclonus, increased tone, and palmomental reflex. She was febrile with elevated CRP and bibasilar opacity on CXR. She was COVID positive on PCR. Head CT was nil acute and MR, EEG, LP were not done. She was trialed on olanzapine and haloperidol for management with poor effect. Switched to chlorpromazine and clonidine patch	Palliative	Very low quality
	(2) AUD—remitted, NCD 70 years Male	Admitted with aggression, staring, alogia, and abulia. On examination had cogwheel rigidity and myoclonus. An EEG showed diffuse slowing and generalized discharges and head CT was nil acute. He was COVID positive on PCR. Lorazepam was trialed for query catatonia with poor effect. His aggression and delirium were managed with physical restraints and valproic acid	Improved	
	(3) Schizophrenia 68 years Male	Admitted with a fall causing subdural hematoma (seen on head CT), UTI, AKI, and hypercalcemia. He was COVID positive on PCR. His longstanding clozapine and lithium were held, after which he developed agitation, alogia, abulia, and disorientation. He had mild tardive dyskinesia on examination. Delirium was managed with physical restraints and slow reintroduction of antipsychotics	Improved	
	(4) MDD with psychosis, NCD 87 years Female	Admitted with agitation, disorientation, and slurred speech. On examination she had myoclonus. She was tachycardic and had elevated CRP and was COVID positive on PCR. Delirium was initially managed with physical restraints and haloperidol, and later with quetiapine	Deceased	
Martinotti 2020 Italy *n* = 6	(5) MDD 61 years Male	Admitted with COVID pneumonia requiring NIMV. Hyperactive delirium managed with Abilify IM. Pre-dose-ICDSC score 6, post-dose score 2	NR	Very low quality
	(6) MDD with psychosis 60 years Male	Admitted with mild COVID pneumonia and fever. Developed hyperactive delirium, delusions of guilt, and suicidal ideation. Delirium managed with Abilify IM. Pre-dose ICDSC score 4, post-dose score 2	NR	
	(7) BD 58 years Male	Admitted with COVID pneumonia requiring MV. Hyperactive delirium managed with Abilify IM. Pre-dose ICDSC score 5, post-dose 2	Recovered	
	(8) MDD 64 years Male	Admitted with COVID pneumonia requiring NIMV. Hyperactive delirium managed with Abilify IM. Pre-dose ICDSC score 5, post-dose score 2	NR	
	(9) BD 67 years Male	Admitted with COVID pneumonia requiring NIMV. Hyperactive delirium managed with Abilify IM. Pre-dose ICDSC score 5, post-dose score 2	NR	
	(10) GAD 71 years Male	Admitted with COVID pneumonia requiring NIMV. Developed hyperactive delirium and persecutory delusions. Delirium managed with Abilify IM. Pre-dose ICDSC score 6, post-dose score 0	Recovered	
Palomar-Ciria 2020 Spain *n* = 1	(11) Schizophrenia 65 years Male	Admitted with 20-day history of bizarre behavior and incoherent speech, as well as new aggression, insomnia, echolalia, and disorientation. Head MRI was performed because of abulia, showing findings of encephalopathy. He had COVID positive antibodies but a negative PCR. He was managed with melatonin, haloperidol, and amisulpride	Improved	Very low quality
Suwan-wongse 2020 United States *n* = 2	(12) BD 67 years Female.	Admitted with disorientation, incoherent speech, AKI, and lithium toxicity (2.3 mmol/L). She was febrile and had bilateral infiltrates on CXR. She was COVID positive on PCR. Her lithium toxicity was managed with fluid resuscitation	Deceased	Very low quality
	(13) ADHD, ASD, BD 18 years Male.	Admitted with altered consciousness, AKI, and lithium toxicity (2.6 mmol/L). He was febrile and tachycardic. His CXR was normal and he was COVID positive on PCR. He was managed conservatively with fluid resuscitation and cessation of lithium with plan to restart as an outpatient	Recovered	

*AKI* Acute kidney injury, *AUD* alcohol use disorder, *ADHD* attention deficit hyperactive disorder, *ASD* autism spectrum disorder, *BD* bipolar disorder, *CRP* C-reactive protein, *CXR* chest X-ray, *CT* computed tomography, *EEG* electroencephalogram, *GAD* generalized anxiety disorder, *ICDSC* intensive care delirium screening checklist, *IM* intramuscular, *LP* lumbar puncture, *MRI* magnetic resonance imaging, *MDD* major depressive disorder, *MV* mechanical ventilation, *NCD* neurocognitive disorder, *NIMV* noninvasive mechanical ventilation, *NR* not reported, *PCR* polymerase chain reaction, *UTI* urinary tract infection

## Results

### Study selection (flow of studies)

**Fig. 1:**
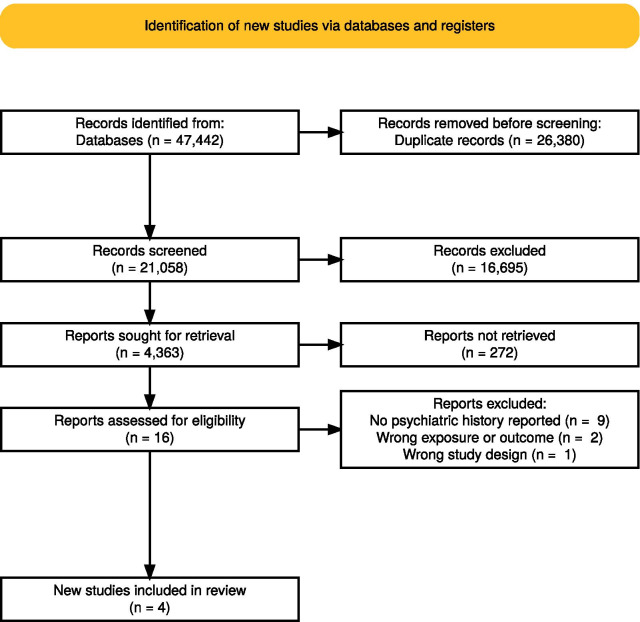
PRISMA flow diagram

Our search for the larger systematic review on the impact of all pandemics and epidemics (including HIV, Zika, SARS, MERS, and so on) on people with preexisting mental disorders yielded 47,442 articles; this decreased to 21,058 after duplicates were removed. After initial title and abstract screening, 4628 reports met criteria for retrieval. After full text retrieval and screening, 669 papers met initial inclusion criteria for extraction. Given the ongoing pandemic, COVID-19 papers were prioritized for extraction, yielding 201 papers consisting of both neuropsychiatric and psychiatric outcomes (refer to our protocol [24] for more information). The psychiatric outcomes review is currently ongoing. In total, only 16 of the 201 COVID-19 studies reported on neuropsychiatric sequelae. These 16 studies were further reviewed for the current paper; 4 papers met final inclusion criteria and 12 were excluded at this stage. No automation tools were used in this process. Our flow diagram (Fig. 1.) was created using the PRISMA 2020 flow tool [30].

### Study selection (excluded studies)

Of the 12 papers excluded during extraction phase, 4 were excluded for not reporting psychiatric history, despite reporting other medical history [31–34]. Other studies were excluded for not reporting any comorbidity data, or for having the wrong exposure (that is, not COVID-19 infection), outcome, or study design.

### Study characteristics

Four studies were included in our review [35–38]. The key characteristics of the included studies are presented in Table 1. Included studies presented data for 23 hospitalized COVID-19 patients, of which 13 (57%) had a previous psychiatric diagnosis. Only data for those with a psychiatric history are presented and discussed. The studies were from high-income countries only, including the United States, Spain, and Italy.

### Risk of bias in studies

A summary of the risk of bias of studies is presented in Table 2. Each item was assigned a score of either low risk of bias (green), unclear risk of bias (yellow), or high risk of bias (red). Two of the tool’s questions were not applicable to our included studies and as such they are not presented in the table. These questions were “Was there a challenge/re-challenge phenomenon” and “Was there a dose-response effect”.

**Table 2:**
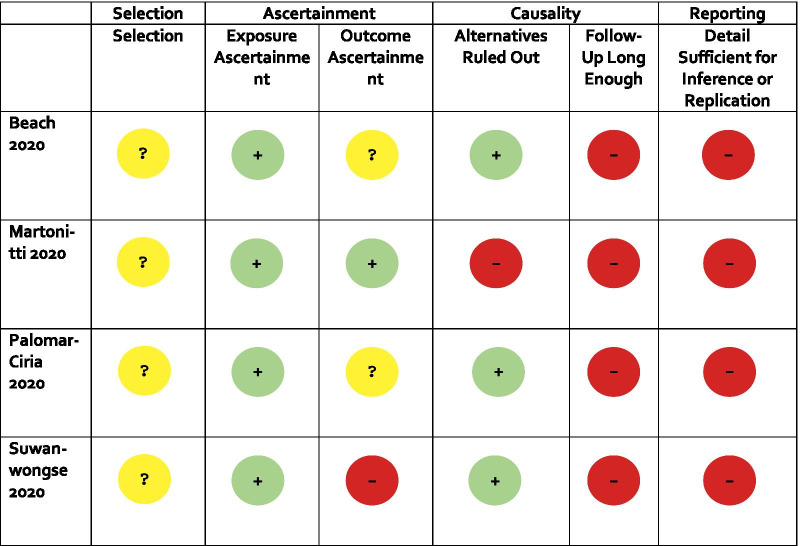
Risk of bias

All included studies were deemed as having unclear risk of bias for selection of cases [35–38], as inclusion and exclusion criteria were never provided. The studies were all at low risk of bias for ascertainment of exposure (COVID-19 infection) as each case reported either findings from a PCR or antibody test, or provided a strong diagnostic rationale based on clinical suspicion [35–38]. The items deemed most relevant to the current study were ascertainment of outcome (neuropsychiatric sequelae) and ruling out alternative causes of the outcome (that is, besides COVID-19). One study clearly specified their diagnostic criteria for delirium, and as such, it was at low risk of bias [36]. The two studies with unclear risk of bias for this variable clearly documented outcomes, although it was not clear what diagnostic criteria, if any, were utilized to make a diagnosis [35, 37]. Finally, one of the papers was at high risk of bias as they used vague terms to describe neuropsychiatric outcomes (for example, behavior change) [38]. All but one study provided alternative explanations for the neuropsychiatric outcomes or ruled out differential etiologies (for example, using neuroimaging or laboratory investigation), as such these were at low risk of bias for this domain [35,^ 37^, 38]. All studies were at high risk of bias due to follow-up; most papers did not specify the duration of their study and none were long enough to allow for a full understanding of how the neuropsychiatric outcome impacted the participant(s), even in the short term [35–38]. Included studies were all at high risk of bias regarding the ability to make inferences to future patients because of insufficient or unclear details provided on the cases [35–38].

### Results of individual studies

We summarize the included studies [35–38] in Table 1. Here we provide the country of origin, sample size, patient descriptions (including psychiatric diagnosis and age), qualitative description of the cases and neuropsychiatric manifestations, and the patient’s eventual disposition.

### Results of synthesis

#### Neuropsychiatric manifestations

In Table 3, we provide a visual summary of the neuropsychiatric outcomes from each case. Of these, the neuropsychiatric manifestation consistently described across studies was delirium. According to the DSM-5 [16], delirium can be sub-specified into hyperactive (involving mood lability, agitation, and/or refusing care), hypoactive (involving sluggishness or lethargy), or mixed. All cases from Martonitti *et al*. [36] explicitly presented hyperactive delirium as the primary clinical picture, and most other cases, except potentially cases 2, 12, and 13, were likely also hyperactive based on their description. Other common manifestations of COVID-19 were agitation or aggression, dysarthria, abulia, and perceptual disturbance. While these signs and symptoms can indicate other underlying medical problems or psychopathology, they are also common features of delirium [39, 40].

**Table 3:** Summary of neuropsychiatric manifestations of COVID-19

	Delirium	Agitation/aggression	Dysarthria	Abulia	Perceptual disturbance	Alogia	Myoclonus	Rigidity
Case 1	+	+	−	+	NR	+	+	+
Case 2	+	+	−	+	NR	+	+	+
Case 3	+	+	−	+	NR	+	−	−
Case 4	+	+	+	−	NR	−	+	−
Case 5	+	+	NR	NR	NR	NR	NR	NR
Case 6	+	+	NR	NR	+	NR	NR	NR
Case 7	+	+	NR	NR	NR	NR	NR	NR
Case 8	+	+	NR	NR	NR	NR	NR	NR
Case 9	+	+	NR	NR	NR	NR	NR	NR
Case 10	+	+	NR	NR	+	NR	NR	NR
Case 11	+	+	+	+	−	−	NR	NR
Case 12	+	NR	+	NR	NR	NR	NR	NR
Case 13	+	NR	−	NR	NR	NR	NR	NR
Tot	13	11	3	3	2	3	3	2

+ enough data provided to infer finding; − enough data to infer lack of finding

*NR* not reported, *Tot* total

Physical examination findings were not reported for most cases, though three cases reported on findings of myoclonus and two of rigidity. Neither myoclonus nor rigidity are included in any of the common tools for characterizing clinical features of delirium [39, 40], suggesting a different etiology. Alogia was also present in three included cases, which similarly is not an associated sign of delirium.

#### Mental disorder type

This review found that people with a number of underlying psychiatric disorders experienced neuropsychiatric sequelae of COVID-19, including mood disorders, alcohol use disorder (AUD), schizophrenia, generalized anxiety disorder (GAD), and neurodevelopmental disorders. None of the studies addressed how the history of psychiatric disorder was diagnosed or confirmed (for example, self report, medical record, diagnostic criteria used). In keeping with prior research on risk factors for delirium [19], mood disorders were the most commonly reported psychiatric comorbidity. In our review, 62% of cases of delirium had a preexisting mood disorder, either major depressive disorder (MDD) or bipolar disorder (BD), prior to COVID-19 infection. Furthermore, 15% of patients were diagnosed with schizophrenia (*n* = 2) and 15% with AUD (*n* = 2) prior to their infection. Although we did not specifically assess for preexisting neurocognitive disorders (NCDs), these diagnoses also pose a heightened risk for delirium, and in a prior systematic review [18], were implicated in 22–89% of delirium cases. Our results are in keeping with this finding, in that NCDs were comorbid in 23% of patients.

#### Age

Older age is an important risk factor for the development of delirium [18, 19]. Almost all patients described in our review were older adults (58–87 years). The outlier among the cases was an 18-year-old who developed features of delirium in the context of COVID-19 infection, as well as lithium toxicity. This individual may have had an underlying brain vulnerability and heightened risk for delirium due to his neurodevelopmental disorders.

#### Management and specific challenges for psychiatric patients

In our review, psychotropic medications were implicated in at least three (23%) cases of neuropsychiatric manifestations of COVID-19. In case 3, lithium and clozapine were abruptly withheld owing to electrolyte disturbances, kidney injury, and COVID-19, all of which may have contributed to the precipitation of delirium. Cases 12 and 13 were similarly multifactorial in that each were delirious in the context of COVID-19, kidney injury, lithium toxicity, and lithium withdrawal. Furthermore, several different management approaches were used in the included studies, including physical restraints and antihypertensives, as well as different psychotropic medications—benzodiazepines, antipsychotics, and mood stabilizers. We are unable to comment on treatment efficacy given the study design of the included papers and lack of long-term follow-up.

### Certainty of evidence

In accordance with the GRADE Framework, all outcomes assessed in this review are considered a very low quality of evidence, given that the derivation of evidence is entirely from case series and reports (Table 1).

## Discussion

### Interpretation

In our review, delirium was the predominant neuropsychiatric sequela identified. The delirium was often accompanied by either agitation or aggression, and occasionally with other associated features [40] such as dysarthria, abulia, and perceptual disturbances. In keeping with emerging literature [41], hyperactive delirium was more common in our review than the hypoactive subtype. In some cases, other confounding etiologies for delirium besides COVID-19 infection were also mentioned, which is in keeping with a multifactorial model of delirium development.

Our review highlights unique challenges for people with psychiatric disorders during COVID-19 delirium. First, psychiatric medications can be deliriogenic [18, 42], however, acute withdrawal of these drugs can also precipitate delirium, which may have contributed to the delirious presentation of at least three cases (3, 12, 13). Second, managing delirium is complicated [43]; the studies in our review employed a variety of medication classes, most of which can be deliriogenic themselves and can cause harmful drug–drug interactions with psychotropics that psychiatric patients may already be treated with. Third, psychiatric disorders can present a diagnostic challenge during delirium states [40] and can also bias clinicians against infectious etiologies as a source for a delirious presentation. Given our findings, we suggest that COVID-19 infection be considered in people with psychiatric disorders who present with an acute mental status change during the ongoing pandemic.

It is crucial for clinicians to be able to recognize and manage delirium during the ongoing pandemic for a number of reasons. First, it may be the presenting feature of COVID-19 infection in people with premorbid vulnerabilities [44] such as psychopathology. Second, while delirium can last for months [45], meta-analytical data show delirium is an independent risk factor for dementia, institutionalization, and death, suggesting that it may precipitate permanent neurological insult in some patients [27]. Indeed, one study found delirium was associated with a five-fold increased risk of mortality [46]. Preliminary data shows that COVID-19 delirium is also associated with poor outcomes, such as functional impairment at 1 month [12] and neurological and psychiatric morbidity at 6 months [14].

Besides delirium and features commonly associated with this disease, there were other occasionally reported findings in our review including rigidity, myoclonus, and alogia. Interestingly, there are other small reports [47, 48] of myoclonus during COVID-19 infection. More data is certainly needed to better understand the potential relationship between myoclonus and delirium and/or myoclonus and COVID-19. While the scope of this paper precludes a full discussion of the many potential causes of these findings, some have postulated these collective findings suggest an underlying catatonic or dopamine-depleted state during COVID-19 infection [43].

The patient dispositions were highly variable among cases presented in our study. Two patients died and one was transitioned to palliative measures. Many of the patients were noted to have improved, however, most of the studies did not document the duration of their study nor provide a follow-up great enough to draw conclusions on long-term outcomes. While long-term symptoms of COVID-19 are increasingly recognized in the literature [49], more research is needed to fully understand this proposed phenomenon. Future researchers should conduct longitudinal studies of neuropsychiatric outcomes in COVID-19 in people with and without psychiatric disorders to understand the potential effect of psychopathology. This is especially important given the potential for permanent neurocognitive changes after delirium [27], the heightened vulnerability due to psychiatric disorders [20], and the greater risk of contracting COVID-19 in psychiatric populations [50].

An unexpected and important finding garnered from our review processes was the incompleteness of the body of evidence. Most notably, many papers either did not report any comorbidity data or reported only on “medical” disorders. For instance, for studies [31–34] were excluded because they reported medical but not psychiatric history. With a lifetime prevalence for mental disorders of up to 50% [51], it is nearly impossible that none of these large-sample papers included people with psychiatric disorders.

Consideration of psychiatric comorbidity appears to be lacking in other aspects of the emerging COVID-19 literature. Neither a large study of factors associated with COVID-19 mortality [52] nor a meta-analysis of comorbidities associated with prognosis [53] provided psychiatric history data. This is especially concerning as, for instance, a link between schizophrenia and higher risk for in-hospital COVID-19 mortality has been established [54, 55] and there is a known all-cause mortality gap for people with psychiatric disorders [56, 57].

The consideration of psychiatric history in papers assessing neurological and psychiatric outcomes of COVID-19 is imperative, given that premorbid psychiatric disease may be an important confounder or a risk factor for poorer outcomes overall [58]. Surprisingly, consideration of psychiatric history appears to have eluded these emerging papers. For instance, a study on 6-month neuropsychiatric outcomes in over 200,000 COVID-19 survivors [10] did not control for preexisting psychiatric disorders, nor examine this population as a separate cohort. Additionally, a recent prospective study on delirium in critical care COVID-19 patients did not mention psychiatric history [41].

The rationale for not collecting or reporting preexisting psychiatric disorders, despite reporting, or controlling for, other medical comorbidities is unclear. It is possible that this is partially contributed to by stigma. It is well known that psychiatric disorders are stigmatized [59, 60], which may lead patients to under report their diagnoses or have precluded researchers from collecting this data. Alternatively, researchers may not have deemed psychiatric disorders an important comorbidity to report in COVID-19 literature for unknown reasons. Moving forward, we encourage researchers to continue to study COVID-19 morbidity and mortality in people with psychiatric disorders and control for past psychiatric history in all emerging COVID-19 research.

### Limitations of evidence and review process

There are several limitations of this review. First, all studies were case reports or series, study designs that prevent any inference of causality and limits interpretation of our findings. Second, the overall sample size was small, which limits the generalizability of results. Third, the follow-up of patients was brief, therefore, conclusions about long-term neuropsychiatric effects of COVID-19 cannot be drawn. Fourth, studies lacked consistency in terms of defining neuropsychiatric outcomes and considering other etiologies for neuropsychiatric sequelae besides COVID-19, as well as reporting on how preexisting psychiatric disorder was diagnosed and confirmed; therefore, the validity of our findings may be limited. Fifth, non-English studies were excluded, and as such, the review may not be completely comprehensive.

## Conclusions/implications

To our knowledge, this is the first study to summarize the literature regarding the neuropsychiatric manifestations of COVID-19 infection in the psychiatric population. The main findings are that people with psychiatric disorders can develop delirium during COVID-19 infection and that there is a dearth of data that report on both neuropsychiatric sequelae and psychiatric history. We also found a variety of medication classes are used to manage delirium, which calls for future studies to evaluate the effectiveness of treatments for neuropsychiatric symptoms in COVID-19. We additionally highlight the need for emerging COVID-19 studies to focus on or sub-analyze by psychiatric history.

## Supplementary Information

**Additional file 1. Table S1.** PRISMA Research Checklist.

**Additional file 2. Table S2.** Search Strategy.

## Data Availability

Data are available upon request to the corresponding author. All data used are also available from the original publications included in this review.

## Abbreviations

CNS: Central nervous system
PCR: Polymerase chain reaction
PRISMA: Preferred reporting items for systematic reviews and meta-analyses
AUD: Alcohol use disorder
NCD: Neurocognitive disorder
CRP: C-reactive protein
CXR: Chest X-ray
CT: Computed tomography
MR: Magnetic resonance
EEG: Electroencephalogram
LP: Lumbar puncture
UTI: Urinary tract infection
AKI: Acute kidney injury
MDD: Major depressive disorder
NIMV: Noninvasive mechanical ventilation
ICDSC: Intensive care delirium screening checklist
NR: Not reported
BD: Bipolar disorder
MV: Mechanical ventilation
GAD: Generalized anxiety disorder
ADHD: Attention deficit hyperactive disorder
ASD: Autism spectrum disorder
Tot: Total
