# Supplementary material for: Delirium and other neuropsychiatric manifestations of COVID-19 infection in people with preexisting psychiatric disorders: a systematic review
Source: J Med Case Rep. 2021 Dec 13;15:586. doi: 10.1186/s13256-021-03140-6 (PMC8667019; doi:10.1186/s13256-021-03140-6)
Supplement: Supplementary file 2 — Additional file 2. Table S2. Search Strategy. [file 13256_2021_3140_MOESM2_ESM.pdf]

Appendix 1. Database Search Strategy

Database	Search Strategy

MEDLINE = 4044	1 (exp coronavirus/ or coronavirus*.mp.) and (wuhan or beijing or shanghai or 2019-nCoV or nCov or COVID-19 or SARS-CoV-2).mp.<br>2 coronavirus*.ti. or (novel coronavirus*.mp. and (exp china/ or china.mp.)) or ((pneumonia.mp. or exp pneumonia/) and Wuhan.mp.)<br>3 ('COVID-19' or '2019-nCov' or 'SARS-CoV-2').mp. or exp Coronavirus Infections/<br>4 Severe Acute Respiratory Syndrome/<br>5 severe acute respiratory syndrom*.ti,ab,kf.<br>6 (sars or sars-cov).ti,ab,kf.<br>7 disease outbreaks/ or exp epidemics/<br>8 (disease outbreak* or pandemic* or epidemic*).ti,ab,kf.<br>9 (mers virus or mers-cov or middle east* respiratory syndrom*).ti,ab,kf.<br>10 Influenza Pandemic, 1918-1919/<br>11 Influenza A Virus, H1N1 Subtype/<br>12 (swine flu or spanish flu or h1n1).ti,ab,kf.<br>13 exp HIV Infections/ or Acquired Immunodeficiency Syndrome/<br>14 (HIV or AIDS or human immunodeficienc* syndrome* or acquired immunodeficiency syndrome*).ti,ab,kf.<br>15 Zika Virus Infection/ or Zika Virus/<br>16 zika.ti,ab,kf.<br>17 Hemorrhagic Fever, Ebola/<br>18 ebola.ti,ab,kf.<br>19 1 or 2 or 3 or 4 or 5 or 6 or 7 or 8 or 9 or 10 or 11 or 12 or 13 or 14 or 15 or 16 or 17 or 18<br>20 (physical distanc* or social distanc* or social support* or home confine* or virtual work* or "working from home" or school closure* or online educat* or online learn* or remote work* or work closure* or virtual health or online health or telemedicine or transportation closure* or travel suspension* or travel closure* or border closure* or mass-media campaign* or office closure* or access to healthcare or access to health services or dentist* closure* or mental health facility shutdown or mental health facility closure* or addiction facility shutdown or addiction facility closure* or park closure* or public event* cancel* or social restrict* or state of emergency or public health emergency or recreational facility closure* or entertainment facility closure* or long-term care closure* or travel restrict*).ti,ab,kf.

	21 Quarantine/ or Telemedicine/ or Health Services Accessibility/<br>22 (quarantine or isolat*).ti,ab,kf.<br>23 Patient Isolation/<br>24 Social Support/<br>25 Travel/ or Air Travel/<br>26 20 or 21 or 22 or 23 or 24 or 25<br>27 (mental illness* or mental disorder* or mental health or anxiety or anxious or schizophren* or posttraumatic stress disorder* or post traumatic stress disorder* or bipolar or major depressi* disorder* or depression or major depressive episode* or postpartum depression or ADHD or attention deficit disorder or attention deficit hyperactivity disorder* or panic disorder* or borderline personality disorder* or mental status).ti,ab,kf.<br>28 19 and 26<br>29 exp Mental Disorders/<br>30 27 or 29<br>31 28 and 30

PsychInfo= 1679	1 (coronavirus* and (wuhan or beijing or shanghai or 2019-nCoV or nCov or COVID-19 or SARS-CoV-2)).mp.<br>2 coronavirus*.ti. or (novel coronavirus* and china).mp. or ((pneumonia.mp. or exp pneumonia/) and Wuhan.mp.)<br>3 ('COVID-19' or '2019-nCov' or 'SARS-CoV-2').mp.<br>4 Severe Acute Respiratory Syndrome/<br>5 severe acute respiratory syndrom*.ti,ab.<br>6 (sars or sars-cov).ti,ab.<br>7 disease outbreaks/ or exp epidemics/<br>8 (disease outbreak* or pandemic* or epidemic*).ti,ab.<br>9 (mers virus or mers-cov or middle east* respiratory syndrom*).ti,ab.<br>10 Influenza Pandemic, 1918-1919/<br>11 Influenza A Virus, H1N1 Subtype/<br>12 (swine flu or spanish flu or h1n1).ti,ab.<br>13 exp HIV Infections/ or Acquired Immunodeficiency Syndrome/<br>14 exp HIV/<br>15 (HIV or AIDS or human immunodeficienc* syndrome* or acquired immunodeficiency syndrome*).ti,ab.<br>16 exp Infectious Disorders/ or exp Viral Disorders/<br>17 zika.ti,ab.<br>18 Hemorrhagic Fever, Ebola/<br>19 ebola.ti,ab.<br>20 1 or 2 or 3 or 4 or 5 or 6 or 7 or 8 or 9 or 10 or 11 or 12 or 13 or 14 or 15 or 16 or 17 or 18 or 19<br>21 (physical distanc* or social distanc* or social support* or home confine* or virtual work* or "working from home" or school closure* or online educat* or online learn* or remote work* or work closure* or virtual health or online health or telemedicine or transportation closure* or travel suspension* or travel closure* or border closure* or mass-media campaign* or office closure* or access to healthcare or access to health services or dentist* closure* or mental health facility shutdown or mental health facility closure* or addiction facility shutdown or addiction facility closure* or park closure* or public event* cancel* or social restrict* or state of emergency or public health emergency or recreational facility closure*

	or entertainment facility closure* or long-term care closure* or travel restrict*).ti,ab.<br>22 Quarantine/ or Telemedicine/ or Health Services Accessibility/<br>23 (quarantine or isolat*).ti,ab.<br>24 Patient Isolation/<br>25 Social Support/<br>26 Travel/ or Air Travel/<br>27 21 or 22 or 23 or 24 or 25 or 26<br>28 20 and 27<br>29 exp Mental Disorders/<br>30 (mental illness* or mental disorder* or mental health or anxiety or anxious or schizophren* or posttraumatic stress disorder* or post traumatic stress disorder* or bipolar or major depressi* disorder* or depression or major depressive episode* or postpartum depression or ADHD or attention deficit disorder or attention deficit hyperactivity disorder* or panic disorder* or borderline personality disorder* or mental status).ti,ab.<br>31 29 or 30<br>32 28 and 31

CINAHL=347	1 (MH "Coronavirus+") OR "coronavirus" OR (MH “Coronavirus Infections+”)<br>2 (MH "Severe Acute Respiratory Syndrome") OR (MH "SARS Virus") OR (MH "Respiratory Distress Syndrome, Acute") OR "Severe Acute Respiratory Syndrome/" OR (MH "Respiratory Distress Syndrome")<br>3 AB severe acute respiratory syndrom*<br>4 (MH "Disease Outbreaks") OR "disease outbreaks"<br>5 "pandemic"<br>6 "epidemic"<br>7 (MH "Middle East Respiratory SyndromeCoronavirus") OR (MH "Middle East Respiratory Syndrome") OR "mers"<br>8 (MH "Influenza, Pandemic (H1N1) 2009") OR (MH "Influenza, Avian") OR (MH "Influenza A Virus") OR (MH "Influenza A H5N1") OR (MH "Influenza, Swine") OR (MH "Influenza, Human") OR "Influenza Pandemic"<br>9 (MH "Influenza A Virus") OR "Influenza A Virus" OR (MH "Influenza A Virus, H1N1 Subtype") OR (MH "Influenza A Virus, H3N2 Subtype") OR (MH "Influenza A Virus, H5N1 Subtype") OR (MH "Influenza A H5N1")<br>10 "swine flu" OR (MH "Influenza, Swine")<br>11 "spanish flu" OR (MH "Influenza")<br>12 (MH "HIV Infections") OR "HIV Infections" OR (MH "Human Immunodeficiency Virus") OR (MH "HIV-Infected Patients")<br>13 (MH "Zika Virus")<br>14 (MH "Zika Virus Infections")<br>15 (MH "Hemorrhagic Fever, Ebola") OR "Hemorrhagic Fever, Ebola" OR (MH "Hemorrhagic Fever with Renal Syndrome") OR (MH "Hemorrhagic Fevers, Viral") OR (MH "Dengue Hemorrhagic Fever") OR (MH "Intracranial Hemorrhage")<br>16 "zika"<br>17 "ebola"<br>18 1 OR 2 OR 3 OR 4 OR 5 OR 6 OR 7 OR 8 OR 9 OR 10 OR 11 OR 12 OR 13 OR 14 OR 15 OR 16 OR 17<br>19 "social distanc*"<br>20 "physical distanc*"<br>21 "home confine*"<br>22 "virtual work*"<br>23 (MH "Telecommuting") OR "virtual work or distance work or remote work or e-work or telework"<br>24 "working from home"

	25 "school closure*"<br>26 "online educat*"<br>27 "online learn*"<br>28 "remote work*"<br>29 "work closure*"<br>30 (MH "Health Facility Closure") OR "virtual health or online health or telemedicine or transportation closure*"<br>31 (MH "Air Travel") OR "travel suspension*"<br>32 "travel closure*"<br>33 "border closure*"<br>34 "long-term care closure"<br>35 (MH "Assisted Living")<br>36 (MH "Quarantine") OR (MH "Social Isolation (Saba CCC)") OR (MH "Social Isolation") OR (MH "Patient Isolation") OR (MH "Social Isolation (NANDA)") OR "quarantine and isolation"<br>37 (MH "Telemedicine")<br>38 (MH "Health Services Accessibility") OR "Health Services Accessibility"<br>39 19 OR 20 OR 21 ORS22 OR 23 OR 24 OR 25 OR 26 OR 27 OR 28 OR 29 OR S30 OR 31 OR 32 OR 33 OR 34 OR 35 OR 36 OR 37 OR 38<br>40 18 AND 39<br>41 (MH "Mental Disorders") OR "mental disorders" OR (MH "Behavioral and Mental Disorders")<br>42 "mental illness"<br>43 "(mental illness* or mental disorder* or mental health or anxiety or anxious or schizophren* or<br>posttraumatic stress disorder* or post traumatic stress disorder* or bipolar or major depressi* disorder* or depression or major depressive episode* or postpartum depression or ADHD or attention deficit disorder or attention deficit hyperactivity disorder* or panic disorder* or borderline personality disorder* or mental status).ti,ab." OR (MH "Mental Disorders, Chronic")<br>44 (MH "Anxiety") OR "anxiety" OR (MH "Anxiety Disorders") OR (MH "Generalized Anxiety Disorder")<br>45 (MH "Depression") OR "depression" OR (MH "Bipolar Disorder") OR (MH "Seasonal Affective Disorder")<br>46 (MH "Stress Disorders, Post-Traumatic") OR "ptsd or post traumatic stress disorder"<br>47 (MH "Schizophrenia") OR "schizophrenia"

	48 (MH "Borderline Personality Disorder") OR "borderline personality disorder" OR (MH "Personality Disorders")<br>49 (MH "Depression, Postpartum") OR "postpartum depression"<br>50 (MH "Panic Disorder") OR "panic disorder"<br>51 (MH "Attention Deficit Hyperactivity Disorder") OR "adhd or attention deficit hyperactivity disorder"<br>52 41 OR 42 OR 43 OR 44 OR 45 OR 46 OR 47 OR 48 OR 49 OR 50 OR 51<br>53 40 AND 52

EMBASE = 2450	1 (exp coronavirus/ or coronavirus*.mp.) and (wuhan or beijing or shanghai or 2019-nCoV or nCov or COVID-19 or SARS-CoV-2).mp.<br>2 coronavirus*.ti. or (novel coronavirus*.mp. and (exp china/ or china.mp.)) or ((pneumonia.mp. or exp pneumonia/) and Wuhan.mp.)<br>3 ('COVID-19' or '2019-nCov' or 'SARS-CoV-2').mp. or exp Coronavirus Infections/<br>4 Severe Acute Respiratory Syndrome/<br>5 severe acute respiratory syndrom*.ti,ab.<br>6 (sars or sars-cov).ti,ab.<br>7 disease outbreaks/ or exp epidemics/<br>8 (disease outbreak* or pandemic* or epidemic*).ti,ab.<br>9 (mers virus or mers-cov or middle east* respiratory syndrom*).ti,ab.<br>10 Influenza Pandemic, 1918-1919/<br>11 Influenza A Virus, H1N1 Subtype/<br>12 (swine flu or spanish flu or h1n1).ti,ab.<br>13 exp HIV Infections/ or Acquired Immunodeficiency Syndrome/<br>14 (HIV or AIDS or human immunodeficienc* syndrome* or acquired immunodeficiency syndrome*).ti,ab.<br>15 Zika Virus Infection/ or Zika Virus/<br>16 zika.ti,ab.<br>17 exp Ebola hemorrhagic fever/<br>18 ebola.ti,ab.<br>19 1 or 2 or 3 or 4 or 5 or 6 or 7 or 8 or 9 or 10 or 11 or 12 or 13 or 14 or 15 or 16 or 17 or 18<br>20 (physical distanc* or social distanc* or social support* or home confine* or virtual work* or "working from home" or school closure* or online educat* or online learn* or remote work* or work closure* or virtual health or online health or telemedicine or transportation closure* or travel suspension* or travel closure* or border closure* or mass-media campaign* or office closure* or access to healthcare or access to health services or dentist* closure* or mental health facility shutdown or mental health facility closure* or addiction facility shutdown or addiction facility closure* or park closure* or public event* cancel* or social restrict* or state of emergency or public health emergency or recreational facility closure* or entertainment facility closure* or long-term care closure* or travel restrict*).ti,ab.

	21 Quarantine/ or Telemedicine/ or Health Services Accessibility/<br>22 (quarantine or isolat*).ti,ab.<br>23 Patient Isolation/<br>24 Social Support/<br>25 Travel/ or Air Travel/<br>26 20 or 21 or 22 or 23 or 24 or 25<br>27 (mental illness* or mental disorder* or mental health or anxiety or anxious or schizophren* or posttraumatic stress disorder* or post traumatic stress disorder* or bipolar or major depressi* disorder* or depression or major depressive episode* or postpartum depression or ADHD or attention deficit disorder or attention deficit hyperactivity disorder* or panic disorder* or borderline personality disorder* or mental status).ti,ab.<br>28 Mental Disorders/<br>29 27 or 28<br>30 19 and 26<br>31 29 and 30

MedRx = 42	“COVID-19” “SARS-CoV-2” “coronavirus” “pandemic” “epidemic” “behaviour” “behavioural response” “social isolation” “mental health” “psychological” “psychiatric” “anxiety” “depression” “distress” “stress” “social distancing” “psychosocial”
